# Prevalence of HIV, STIs, and Risk Behaviors in a Cross-Sectional Community- and Clinic-Based Sample of Men Who Have Sex with Men (MSM) in Lima, Peru

**DOI:** 10.1371/journal.pone.0059072

**Published:** 2013-04-25

**Authors:** Amaya G. Perez-Brumer, Kelika A. Konda, H. Javier Salvatierra, Eddy R. Segura, Eric R. Hall, Silvia M. Montano, Thomas J. Coates, Jeff D. Klausner, Carlos F. Caceres, Jesse L. Clark

**Affiliations:** 1 Department of Medicine, Division of Infectious Diseases and Center for World Health, David Geffen School of Medicine at University of California at Los Angeles, Los Angeles, California, United States of America; 2 Unidad de Salud Sexual y Derechos Humanos, Universidad Peruana Cayetano Heredia, Lima, Peru; 3 U.S. Naval Medical Research Unit 6, Lima, Peru; Asociacion Civil Impacta Salud y Educacion, Peru

## Abstract

**Background:**

Further research is necessary to understand the factors contributing to the high prevalence of HIV/STIs among men who have sex with men (MSM) in Peru. We compared HIV/STI prevalence and risk factors between two non-probability samples of MSM, one passively enrolled from an STI clinic and the other actively enrolled from community venues surrounding the clinic in Lima, Peru.

**Methods:**

A total of 560 self-identified MSM were enrolled between May-December, 2007. 438 subjects enrolled from a municipal STI clinic and 122 subjects enrolled during community outreach visits. All participants underwent screening for HIV, syphilis, HSV-2, gonorrhoea, and chlamydia and completed a survey assessing their history of HIV/STIs, prior HIV testing, and sexual behavior.

**Results:**

HIV prevalence was significantly higher among MSM enrolled from the clinic, with previously undiagnosed HIV identified in 9.1% compared with 2.6% of community participants. 15.4 % of all MSM screened were infected with ≥1 curable STI, 7.4% with early syphilis (RPR≥1∶16) and 5.5% with urethral gonorrhoea and/or chlamydia. No significant differences between populations were reported in prevalence of STIs, number of male sex partners, history of unprotected anal intercourse, or alcohol and/or drug use prior to sex. Exchange of sex for money or goods was reported by 33.5% of MSM enrolled from the clinic and 21.2% of MSM from the community (p = 0.01).

**Conclusions:**

Our data demonstrate that the prevalence of HIV and STIs, including syphilis, gonorrhoea, and chlamydia are extremely high among MSM enrolled from both clinic and community venues in urban Peru. New strategies are needed to address differences in HIV/STI epidemiology between clinic- and community-enrolled samples of MSM.

## Introduction

The high prevalence of HIV, syphilis, and HSV-2 infections among men who have sex with men (MSM) in Peru has been well established in previous research [1]–[7], but additional detail is needed to understand risks for infection, and the reach of existing HIV/STI control measures among Peruvian MSM both actively linked to clinical services and those who are not. Previous research has addressed the importance of including both community and clinic samples in HIV/STI epidemiologic surveillance studies, emphasizing that recruitment from a single venue type may yield a distorted perception of risk behaviors and disease prevalence [1]–[6], [8]–[12]. In Peru, previous surveillance studies of MSM based on both passive recruitment of participants from clinic venues as well as active recruitment of participants from community venues with further referral to STI clinics or sentinel sites have found high prevalence of HIV and STIs [1], [6], [7]. Similarly, Cabello and colleagues identified a high prevalence of HIV during community-based outreach testing in Lima (unpublished data). However, there are insufficient data to assess whether substantial differences exist in HIV/STI prevalence between clinic- and community-based samples and to evaluate how accurately clinic-based populations reflect the prevalence of disease and risk behaviors in the larger MSM population.

Analysis of clinic and community samples can also help to assess the impact of existing public health systems in diagnosing new cases of HIV and STI. Peru’s STI control system is based on two key components: *Centros de Referencia de ITS* (STI Referral Centers, or “CERITS”), specialized clinics that provide enhanced prevention and treatment services to MSM, female sex workers, and other at-risk populations; and *Promotores* (Promoters), peer educators who provide HIV/STI information to local communities and encourage at-risk MSM to seek testing at CERITS clinics. *Promotores* are linked to a specific CERITS clinics and often escort patients from the community to clinic sites, although HIV/STI testing services are not routinely provided outside of clinic venues. As a result, comparing the prevalence of HIV/STIs and sexual risk behavior between MSM enrolled from clinic and community venues can both improve understanding of local HIV/STI epidemiology and contribute to an assessment of the reach of clinic-based HIV/STI control systems in detecting new infections within the larger MSM population. In a secondary analysis of data collected for a study of HIV/STI epidemiology among MSM in Peru, we compared the prevalence of HIV/STIs, risk behaviors, and HIV testing practices among MSM passively enrolled from a municipal STI clinic in Lima and participants actively enrolled from community venues in surrounding neighborhoods.

## Methods

### Study Design, Population and Enrollment

We conducted a cross-sectional study of the prevalence of HIV/STIs, sexual risk behavior, and HIV testing history among MSM enrolled from community and clinic venues in Peru. Enrollment was limited to patients born anatomically male who reported oral and/or anal sexual contact with a male or transgender partner in the previous 12 months.

For enrollment from the clinic site, all male and transgender patients visiting the CERITS “Alberto Barton” in Callao were given a flyer explaining the study and asking them to inform staff if they were interested in participating. Counselors screened potential participants for eligibility during routine behavioral counseling sessions.

For enrollment from community venues, participants were identified during testing campaigns conducted in neighborhoods surrounding the CERITS “Alberto Barton.” Hair salons, video arcades and sports areas frequented by MSM were used as temporary sites for enrollment, interviewing, and HIV/STI testing. During outreach campaigns, groups of two or three *Promotores* circulated through the designated community during late afternoon or early evening hours in a van equipped with a public address system to announce the availability of free HIV/STI testing in the neighborhood and the location of the temporary testing venue for that day. Posters and flyers advertising the testing campaign were posted in local venues and distributed to individuals in public spaces.

### Data Collection

All participants completed a survey in Spanish assessing their history of HIV/STIs, sexual risk behavior, and HIV testing practices. Participants responded to questions about socio-demographic factors, sexual behavior (both during the past 6 months and during their last sexual encounter), history of STIs (including prior HIV testing), substance use and transactional sex. Participants in the clinic sample completed the questionnaire independently using private computer stations. Participants enrolled at community venues completed a paper survey independently and in private at the temporary recruitment site. Blood and urine specimens were collected for STI screening, and participants asked to return to the CERITS Barton in one week for delivery of results. Participants diagnosed with a curable STI were given appropriate antibiotic treatment and encouraged to notify recent sex partners of their diagnosis. Participants newly diagnosed with HIV infection were referred to designated Ministry of Health facilities for ongoing care. Monetary incentives were not provided, though participants were offered free condoms and lubricant after completion of the study. Specific procedures to avoid duplicate participation were not conducted during data collection; however, participant date of birth was collected and data was checked for repeat participants as part of the data cleaning prior to data analysis.

All participants provided written informed consent. The Institutional Review Boards of the University of California Los Angeles, the Universidad Peruana Cayetano Heredia, and the U.S. Naval Medical Research Center approved the study protocol in compliance with all federal regulations regarding the protection of human subjects.

### Laboratory Methods

Biological samples were analyzed at the US Naval Medical Research Center Detachment in Lima, Peru. Blood was screened for HIV by EIA (Vironostika, Biomérieux; Marcy l’Étoile, France) with Western Blot confirmation (Genetic Systems, Biorad; Hercules, CA), and for syphilis by rapid plasma reagin (RPR) assay (RPRnosticon, Biomérieux; Marcy l’Étoile, France) with Treponema Pallidum Particle Agglutination (TPPA) confirmation (Serodia, Fujirebio; Tokyo, Japan). TPPA-reactive samples were serially diluted to quantify RPR titers, with early syphilis defined as an RPR≥1∶16. Samples were tested for antibodies to HSV-2 (HerpeSelect, Focus Technologies; Cypress, CA) with a cut-off value for positive results of ≥3.5. Urine specimens were tested for the presence of gonorrhoea/chlamydia using Roche PCR (Cobas Amplicor, Roche Diagnostics; Palo Alto, CA). Rectal and pharyngeal swab specimens were also collected for the diagnosis and treatment of gonorrhea and chlamydia. Due to subsequent findings regarding the poor reliability of the Cobas Amplicor PCR for diagnosis of gonorrhea and chlamydia in extragenital sites, we have omitted those results from the data presented.

### Data Analysis

Statistical analysis compared HIV/STI prevalence, self-reported sexual risk behavior, and HIV testing history between community- and clinic-enrolled samples. Categorical variables were analyzed using chi-squared or Fisher’s exact tests and continuous variables assessed using Kruskal-Wallis or Student’s t-tests. Individuals with missing data were excluded from the affected analysis only. All statistics were calculated with 95% confidence intervals. Stata 11.0 software was used for all analyses (Stata Corporation, College Station, TX).

## Results

We enrolled a total of 560 male or male-to-female transgender individuals reporting sexual contact with a male or transgender partner within the preceding 12 months. 438 participants were enrolled from the CERITS Barton and 122 participants were enrolled during outreach visits to surrounding neighborhoods (See: Table 1).

**Table 1:** Demographics, Prevalence of HIV, STIs, and Risk Behaviors in a Cross-Sectional Community-and Clinic-based Sample of Men Who Have Sex with Men in Lima, Peru; 2007.

	Recruitment Population			
	Combined n = 560 (%)	Clinic n = 438 (%)	Community n = 122 (%)	p-value
**Demographics**				
** Median Age ± IQR**	**28 (23–35)**	**28 (23–36)**	**26 (20–33)**	**0.017**
Graduated High School	435 (78.2)	339 (77.6)	96 (80.7)	0.468
**Sexual Identity**				**0.033**
Homosexual	198 (37.2)	156 (37.6)	42 (35.6)	
Bisexual	60 (11.3)	53 (12.8)	7 (5.9)	
Transgender	21 (22.7)	97 (23.4)	24 (20.3)	
Heterosexual	154 (28.9)	109 (26.3)	45 (38.1)	
**Sex Role**				0.118
* Activo* (Insertive)	170 (32.0)	124 (29.9)	46 (39.3)	
* Pasivo* (Receptive)	179 (33.7)	141 (34.0)	38 (32.5)	
* Moderno* (Versatile)	183 (34.4)	150 (36.1)	33 (28.2)	
**STI Symptoms**				
** Dysuria, Pus, Ulceration (Urethral)**	**154 (27.9)**	**134 (30.8)**	**20 (17.0)**	**0.003**
** Pus, Bleeding, Ulceration, and/or Pain (Rectal)**	**188 (34.2)**	**160 (37.1)**	**28 (23.7)**	**0.007**
**HIV and STI Prevalence**				
** HIV**	**125 (22.3)**	**115 (26.3)**	**10 (8.2)**	**<0.001**
Syphilis (RPR≥1∶16)	40 (7.4)	32 (7.5)	8 (6.7)	0.766
HSV-2	310 (55.4)	248 (56.6)	62 (50.8)	0.254
Urethral Gonorrhoea	12 (2.1)	11 (2.5)	1 (0.8)	0.254
Urethral Chlamydia	23 (4.1)	16 (3.7)	7 (5.7)	0.305
**Sexual Risk Behavior**				
Any Unprotected Anal Intercourse (6 Months)	220 (40.1)	177 (41.4)	43 (35.2)	0.494
Median Number of Male Sex Partners ± IQR (6 Months)	2 (1–6)	2 (1–6)	2 (1–7)	0.787
** Exchange of Sex for Money/Goods (6 Months)**	**168 (30.8)**	**143 (33.5)**	**25 (21.2)**	**0.01**
Use of Alcohol Prior to Sex (1 Month)	291 (53.9)	229 (54.3)	62 (52.5)	0.74
Use of Drugs Prior to Sex (3 Months)	84 (15.3)	72 (16.6)	12 (10.2)	0.084

Notes: Statistical significance for categorical variables was determined using chi-squared or Fisher’s exact tests and continuous variables assessed using Kruskal-Wallis or Student’s t-tests. Variables with missing data were calculated on available data. Variables in bold indicate that the comparison was statistical significant.

### Prevalence of HIV/STIs

**Figure 1:**
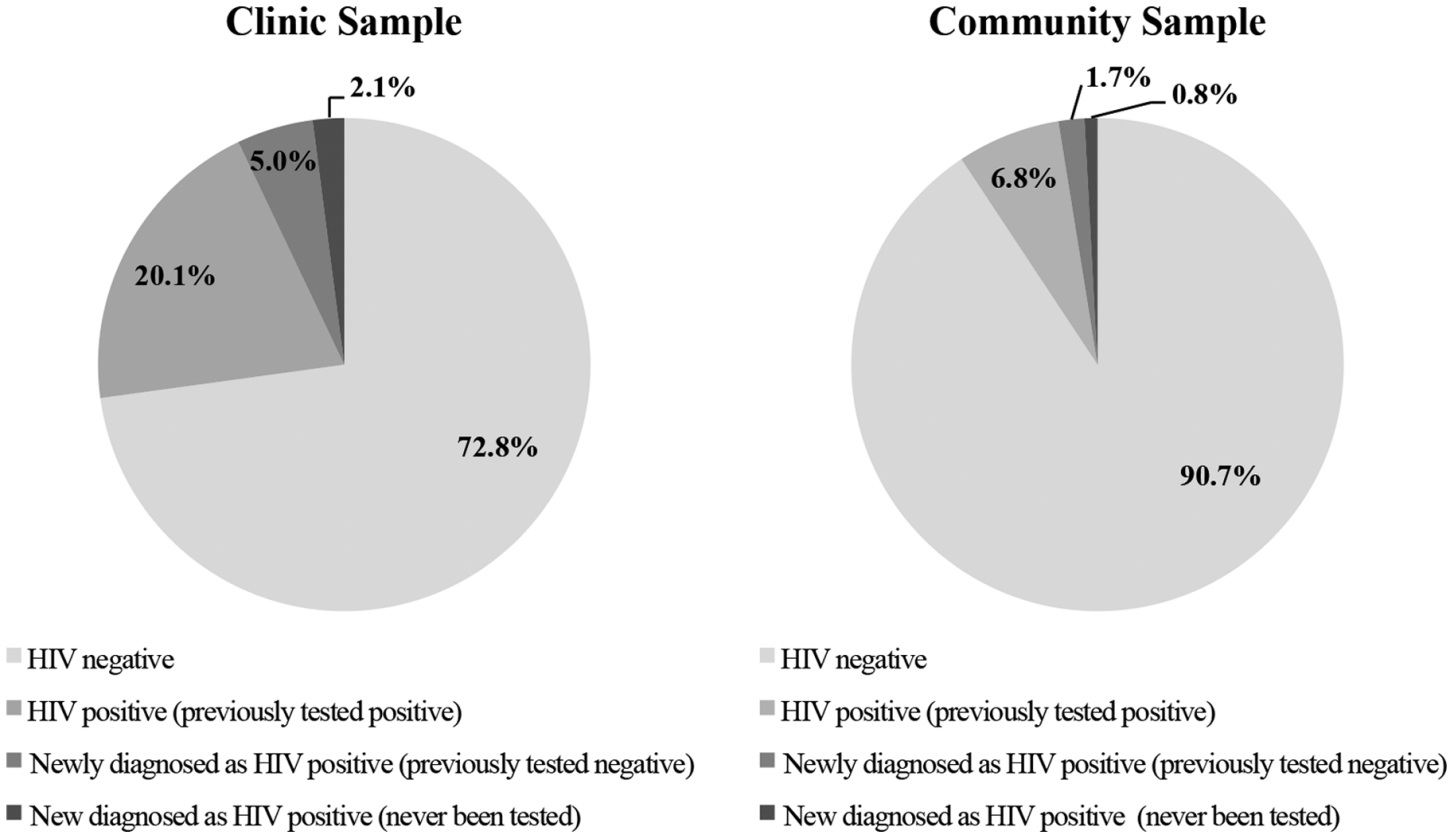
Distribution of HIV Testing History for all MSM from the Clinic (n = 438) and Community-Based Sample (n = 122); Lima, Peru 2007.

HIV prevalence was significantly higher among clinic recruits (26.3% vs. 8.2%; p<0.01). Prior HIV testing was reported by 85.6% of MSM from the clinic and 79.6% from the community (p = 0.11), with 72.4% of all participants having been tested for HIV within the past year. Among participants who had never been tested for HIV, 14.5% (9/62) of MSM from the clinic and 4.2% (1/24) from the community were HIV-positive (p = 0.27). Among MSM who reported that their last HIV test was negative, 7.7% (22/284) of those from the clinic sample and 2.3% (2/86) from the community sample were newly diagnosed as HIV-positive during the study (p = 0.08) (Figure 1).

Within the entire study population, 15.4% of participants were infected with a curable STI: 7.4% with early syphilis (RPR≥1∶16) and 5.5% with urethral gonorrhoea and/or chlamydia. Although clinic patients more commonly reported dysuria and urethral discharge, there was no significant difference in the combined prevalence of laboratory-diagnosed urethral gonorrhea and/or chlamydia (5.5% in clinic sample and 5.8% in the community, p = 0.90). Prevalence of syphilis and HSV-2 were similar between the two groups.

### Sexual Risk Behavior

No significant differences were noted between the two populations in terms of the reported number of male sex partners, history of receptive and/or insertive unprotected anal intercourse, or alcohol and/or drug use prior to sex. Exchange of sex for money or goods was reported by 33.5% of MSM enrolled from the clinic and 21.2% of MSM from the community (p = 0.01).

## Discussion

We present data comparing the prevalence of HIV/STIs, sexual risk behaviors, and HIV testing history between populations of MSM enrolled in community and clinic venues in Lima. We found high prevalence of HIV, syphilis, and HSV-2, and high frequencies of unprotected anal intercourse as well as alcohol use prior to sex among MSM from both clinic- and community-based samples. Only the prevalence of HIV infection was significantly different between the two populations.

Our comparison finds that the prevalence of both established and previously undiagnosed HIV infection was significantly higher in the clinic sample. Although the majority of MSM surveyed had previously been tested for HIV, 7. 1% of MSM from the clinic population and 2.5% of MSM from the community had previously undiagnosed HIV infection. In addition, 40.1% of all MSM surveyed had recently engaged in unprotected anal intercourse and 7.4% had serologic evidence of early syphilis infection. Though the prevalence of chronic STIs like HIV, HSV-2, and previously treated syphilis infection is likely to be higher among clinic participants who have been integrated into existing health care systems, these results point to the partial reach of current efforts for detecting new cases of HIV infection among MSM since current HIV/STI control efforts do not routinely include community-based testing. Yet, similar results between the two samples of MSM in regards to sexual risk behavior, self-perception of HIV risk, and prevalence of STIs indicate the need to further investigate the incorporation of community-based testing to extend the reach of Peruvian HIV/STI control efforts.

Several factors differentiate our findings from previous research in Peru. In contrast to previous studies that have been limited to behaviorally high-risk, HIV-negative or unknown serostatus MSM, enrollment in our sample was open to all sexually active MSM, regardless of HIV status or risk for infection [13]. As a result, our findings provide additional information on the frequency of sexual risk behavior and disease prevalence within the entire MSM population, contributing to greater understanding of the epidemiology of HIV/STIs in the area. Given the recruitment methods used and that our data is derived from convenience samples of MSM enrolled from a single STI clinic and surrounding neighborhoods, caution should be exercised when generalizing our findings to other MSM populations in Peru or Latin America. Importantly, peer health outreach workers, or *Promotores* conducted recruitment for both venues. While this approach is consistent with existing STI control systems in Peru, identification of community venues and participant recruitment may have been limited by individual *Promotores’* knowledge of local MSM populations and/or venues, leading to an underrepresentation of “hidden” populations such as non-gay-identified MSM. The self-selecting nature of both clinic and community samples and that the majority of our participants were enrolled from an STI clinic site, may have led us to overestimate the prevalence of sexual risk behavior and HIV/STIs in the MSM population. Data collection methods varied due to logistical challenges in the community venues and errors may have occurred when transferring paper responses to the electronic database. In addition, there is a possibility that some of the community venue participants may have also been patients of the STI clinic, increasing the likelihood of similarities between sample populations. Despite these limitations, our findings provide evidence supporting the use of mixed-venue recruitment techniques and clinic-to-community comparisons for assessing HIV/STI epidemiology and control measures among MSM in Peru.

Our data shows that MSM enrolled from both STI clinic and community venues in Lima, Peru have high prevalence of HIV infection (both established and previously undiagnosed), STIs (including syphilis and HSV-2), and risk behaviors associated with HIV/STI transmission (including unprotected anal intercourse and alcohol use prior to sex). Our findings provide support for current public health systems using community peer outreach and clinic testing sites to identify new cases of HIV infection, but they also indicate an urgent need for additional prevention efforts to reduce sexual risk behavior and control STI co-infection among MSM in urban Peru. Additional research addressing differences in the epidemiology of HIV/STI transmission within clinic- and community-enrolled samples of MSM is needed to further develop HIV/STI prevention interventions in Peru.
